# Enuresis in Early Adolescents

**Published:** 2017-10

**Authors:** Sevim SAVASER, Nezihe KIZILKAYA BEJI, Ergul ASLAN, Duygu GOZEN

**Affiliations:** 1. Division of Child Development, Faculty of Health Sciences, Biruni University, Istanbul, Turkey; 2. Division of Nursing, Faculty of Health Sciences, Biruni University, Istanbul, Turkey; 3. Dept. of Women Health and Diseases Nursing, Florence Nightingale Faculty of Nursing, Istanbul University, Istanbul, Turkey; 4. Dept. of Pediatric Nursing, Florence Nightingale Faculty of Nursing, Istanbul University, Istanbul, Turkey

## Dear Editor-in-Chief

The International Children’s Continence Society defines enuresis as bed-wetting while asleep/nocturnal incontinence (1). About 5–7 million children aged 7 or over were affected by enuresis, and prevalence increased in boys and with enuretic family history (2). Prevalence drops with age (3,4) and decreases to 1%–3% at the age of 15 (5). While enuresis does not result in serious physical discomfort in childhood, it is a problem with adverse effects on the quality of life of many children and their parents due to its social and psychological implications (6, 7). This study was conducted to establish urinary incontinence (UI) in early adolescent aged 11–14, and identify the emotions and social problems of enuretic children. The population of the survey consisted of students from the 5^th^, 6^th^, 7th and 8^th^ grades of public primary schools in Istanbul. The size of the study sample was determined as n=483 for each grade. Overall, 1932 students must be contacted for the sample group, as a minimum requirement. The questionnaires were distributed within the same day in each of the designated schools to be completed in a classroom setting. All volunteering students were enrolled in the study. Evaluation was made on 2750 questionnaires. Data were analyzed with SPSS 15.0 package program (Chicago, IL, USA). *P*-values <0.05 were considered statistically significant.

The mean age of enrolled 2750 early adolescent was 12.53±1.12. 8.6% of early adolescent had UI, based on their own personal statements. Enuresis was more common among early adolescents who reported frequent urinary infections, constipation, and school toilet avoidance. Average of scores given by students who reported UI to the question “How much does UI affect your daily life” was 2.95±2.73 on the Visual Analogue Scale (0–10). Two-thirds of enuretic early adolescents stated that their daily life was slightly and moderately affected by enuresis.

The prevalence of UI was found to be 8.6%, to become less common with age, and to be considerably high in boys and those who reported frequent urinary infections, history of childhood enuresis in the immediate family and a low socioeconomic status.

School and field screening programs should be conducted with school age children and information should be gathered from children and parents for the purposes of diagnosing undisclosed UI in the society, and guidance must be provided about the optimal treatment of children with UI.
